# Pulsatile flow drivers in brain parenchyma and perivascular spaces: a resistance network model study

**DOI:** 10.1186/s12987-018-0105-6

**Published:** 2018-07-16

**Authors:** Julian Rey, Malisa Sarntinoranont

**Affiliations:** Department of Mechanical and Aerospace Engineering, University of Florida, PO Box 116250, Gainesville, FL 32611 USA

**Keywords:** Rat cerebral cortex, Biotransport, Glymphatic theory, Extracellular flow, Bulk flow, Interstitial flow, Lumped parameter, Porous media, Cerebrospinal fluid, Fluid mechanics, Diffusion

## Abstract

**Background:**

In animal models, dissolved compounds in the subarachnoid space and parenchyma have been found to preferentially transport through the cortex perivascular spaces (PVS) but the transport phenomena involved are unclear.

**Methods:**

In this study two hydraulic network models were used to predict fluid motion produced by blood vessel pulsations and estimate the contribution made to solute transport in PVS and parenchyma. The effect of varying pulse amplitude and timing, PVS dimensions, and tissue hydraulic conductivity on fluid motion was investigated.

**Results:**

Periodic vessel pulses resulted in oscillatory fluid motion in PVS and parenchyma but no net flow over time. For baseline parameters, PVS and parenchyma peak fluid velocity was on the order of 10 μm/s and 1 nm/s, with corresponding Peclet numbers below 10^3^ and 10^−1^ respectively. Peak fluid velocity in the PVS and parenchyma tended to increase with increasing pulse amplitude and vessel size, and exhibited asymptotic relationships with hydraulic conductivity.

**Conclusions:**

Solute transport in parenchyma was predicted to be diffusion dominated, with a negligible contribution from convection. In the PVS, dispersion due to oscillating flow likely plays a significant role in PVS rapid transport observed in previous in vivo experiments. This dispersive effect could be more significant than convective solute transport from net flow that may exist in PVS and should be studied further.

## Background

Since the 1970s the perivascular spaces (PVS) surrounding blood vessels have been thought to play a role in solute transport through brain tissue, specifically as conduits for rapid transport [1, 2]. The PVS are extracellular spaces formed by cylindrical arrangements of glial cells that surround intracortical arterioles and veins [3]. Rennels et al. [2] and more recently Iliff et al. [4] found that tracers injected into the subarachnoid space (SAS) of animal models were preferentially transported through the PVS of intracortical arteries at rates faster than would be expected from diffusion alone. In these studies, tracer moved in the direction of blood flow. Ichimura et al. [5] injected fluorescently labeled albumin into cortical perivascular spaces of rats with an open cranial window preparation and using video-densitometric measurements described slow oscillatory tracer motion within the PVS that was not biased in either direction. Carare et al. [6] and more recently Morris et al. [7] observed tracers injected into the parenchyma quickly located in the basal lamina of capillaries and moved through the basal lamina of arterioles opposite the direction of blood flow. Other recent experiments have confirmed observations of rapid tracer transport via PVS [8, 9]. In humans, cerebrospinal fluid (CSF) tracers have been found along the large leptomeningeal arterial trunks with MRI [10]. Together, these findings suggest that a network of intramural and extravascular channels may serve as a means for facilitated transport of dissolved compounds and exchange between interstitial fluid (ISF) and CSF. As such, it may substitute for an absent lymphatic vessel network in the parenchyma by collecting excess ISF and metabolic wastes [11]. Insights into Alzheimer’s disease, Parkinson’s disease, hydrocephalus, and other neurological diseases may be predicated on a precise understanding of how these solute and fluid transport pathways malfunction.

Despite discrepancies in the literature with regard to the direction of solute transport and the anatomical structures involved, strong correlation with vascular pulsatility is a point of agreement [12]. Pulsatility refers to the periodic changes in blood vessel volume caused by heart contractions. The rate of imaging tracer transport from the SAS into the PVS of penetrating arterioles has been positively correlated with arterial pulsatility in animal models [2, 13]. Clearance of beta-amyloid from the parenchyma of mice [13] and of liposomes introduced by intraparenchymal convection enhanced delivery [14] both decreased with decreased pulsatility. Rapid tracer localization within the capillary basal lamina ceased shortly after animal sacrifice [6]. The rate of transport in PVS and its apparent relationship with pulsatility suggests convective transport generated by pulsatility is involved. Convection is here defined as solute transport along with the net flow of its solvent fluid. A number of investigators have developed pulsatility models for fluid flow in the PVS. Coloma et al. [15] and Sharp et al. [16] have examined vascular reflection waves and unsteady PVS hydraulic resistance as drivers of net fluid flow within the PVS, specifically the arterial basement membranes. However, Asgari et al. [17] simulated flow in the PVS due to vascular pulse wave propagation using computational fluid dynamics (CFD) and observed oscillating flow was 10^3^ times greater that net axial flow, evidence against net convective solute transport by peristalsis.

Iliff et al. [4] proposed the glymphatic theory in which CSF enters the PVS surrounding cortical arteries and flows through parenchyma while convectively transporting metabolic wastes to the PVS surrounding veins from which they are ultimately cleared. Astrocytic endfeet expressing AQP4 at the PVS boundary were proposed to play an essential role in this process. Subsequent computational models and experiments have sought to test the glymphatic theory and have challenged many of its tenets, particularly that solutes are transported via convection in the parenchyma [8, 9, 17–19].

Asgari et al. [20] modeled fluid motion through and around astrocytes in the parenchyma with a hydraulic resistance network. Fluid was driven by a constant pressure difference between arterial and venous perivascular spaces and resistances were varied to simulate the effect of AQP4 knockout and increased extracellular volume. More recently, this group has addressed whether arterial pulsatility modeled with CFD produced bulk flow in parenchyma and argued diffusion dominates solute transport there [17]. Jin et al. [18] and Holter et al. [19] imposed pressure differences between arterial and venous PVS in porous media CFD models and concluded solute transport in parenchyma can be explained by diffusion alone.

In this study, a one vessel and two vessel hydraulic network model was developed to explore how pulsatility may drive fluid motion within cortical PVS and parenchyma of the rat. The one vessel model parameters such as pulse amplitude, PVS size, and tissue hydraulic conductivity were varied to predict their effect on fluid motion and solute transport. A two vessel model was also developed to study the effect of pulse amplitude and timing differences between arteries and veins in proximity. A 2D resistance network is a simple tool that captures the essential physics involved, reveals the effect of varying tissue properties, and can help validate future CFD models. Unlike previous resistance network and CFD models [17–20], the present model predicts fluid motion in the PVS and parenchyma together and does not assume a pressure gradient between the arterial and venous PVS, but is instead based on observed changes in vessel diameter during the cardiac cycle. How the predicted fluid motion may result in previously reported tracer transport patterns is discussed.

## Methods

**Fig. 1:**
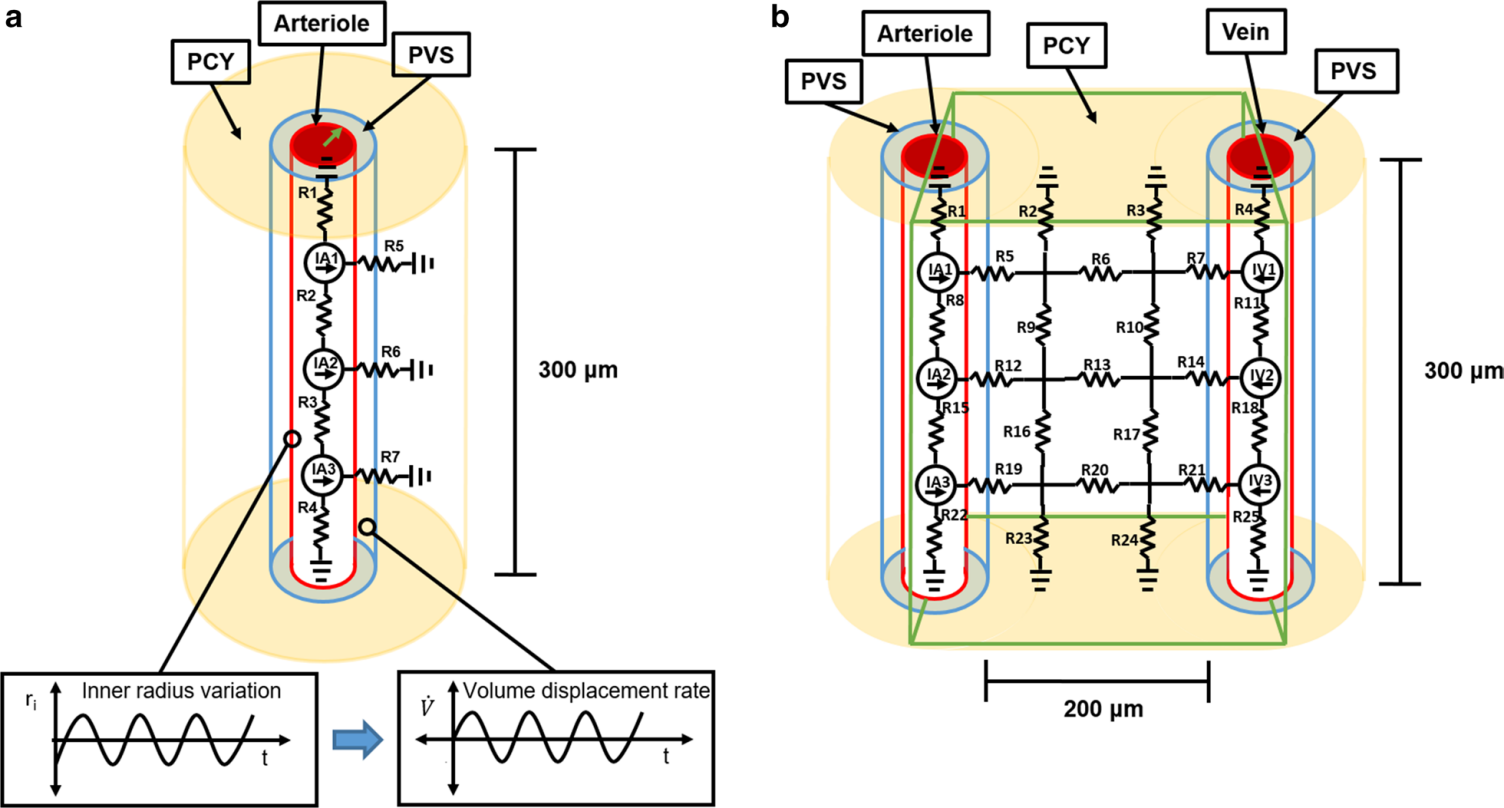
One vessel and two vessel geometries and resistance networks. **a** One vessel model diagram showing the modeled section of a cortical arteriole and its surrounding PVS and parenchyma. The hydraulic resistors are labeled R# and the volumetric fluid sources are labeled IA#. The graphs allude to how PVS inner radius (green arrow) variation displaces fluid volume into the PVS and parenchyma at a certain flow rate (Eqs. 4 and 5). **b** Two vessel model diagram showing the modeled region (green rectangle) of a hypothetical cortical slice containing an arteriole and vein. The hydraulic resistors are labeled R# and the volumetric fluid sources are labeled IA# and IV#

Two hydraulic network models of the PVS and surrounding parenchyma in rat cortex were developed to simulate the fluid motion produced by vascular pulsations: a one vessel model of an arteriole segment, and a two vessel model of arteriole and vein segments (Fig. 1). The vessel segment length and separation were 300 and 200 μm, respectively, which are comparable to mean values found in the literature [19, 21]. Fluid motion through the resistors in the network was governed by the hydraulic equivalent of Ohm’s law.

1 $$\Delta p = Rq$$

where *Δp* is the pressure difference across the resistor, *q* is the volumetric flow rate through the resistor, and *R* is the reciprocal of the hydraulic conductivity, or the hydraulic resistance. The one and two vessel models were implemented and run in MATLAB R2018a (MathWorks^®^, Natick, MA).

### One vessel model

A cylindrical segment of a penetrating arteriole with a baseline radius of 10 μm [4] and its surrounding PVS and parenchyma were modeled as a network with seven resistors (Fig. 1a). Fluid could enter or leave the network axially through the modeled PVS or radially through the parenchyma. Here the PVS was simply considered a low resistance pathway around the vessel that included the basement membrane of smooth muscle cells [7], the space between the vessel and pial sheath, and the space between the pial sheath and the glia limitans. The existence of true spaces between these membranes is debated [7, 22], but a broad description of PVS as is adopted here was provided in a review by Abbott et al. [3] and reflects uncertainty about what spaces are involved in rapid tracer transport and communication between these spaces. This model did not explicitly model aquaporins on the astrocytic endfeet surrounding the PVS but accounts for their effect as a change in parenchyma hydraulic conductivity.

The PVS hydraulic resistance was derived from the Navier–Stokes solution for steady pressure-driven flow through a straight annulus [23].

2 $$R_{PVS} = \frac{8\mu l}{{\pi R_{o}^{4} \left[ {1 - E^{4} + \frac{{\left( {E^{2} - 1} \right)^{2} }}{lnE}} \right]}}$$

Here *μ*, *l*, *R*_*o*_, and *E* are the fluid dynamic viscosity, the PVS length modeled by the resistor, the PVS outer radius, and the ratio of PVS inner to outer radius, respectively. Parameters and their values are listed in Table 1. Because the PVS is a complex physiological space occupied by proteins and other molecules, this hydraulic resistance was considered a lower bound for hydraulic resistance in vivo.

**Table 1:** One vessel and two vessel model parameters

Symbol	Description	Baseline value	Simulated range	Source
*R*_*i*_	PVS inner radius	10 μm	1–29 μm	[4, 13, 21]
*R*_*o*_	PVS outer radius	30 μm	2–30 μm	[4]
L_PVS_	PVS segment length	300 μm	–	[17, 21]
L_PCY_	Distance between vessels	200 μm	–	[19]
*b*	Wave amplitude	0.25 μm	0–0.37 μm	[13]
*f*	Pulse frequency	5 Hz	–	[14]
–	Wave speed	1 m/s		[24–26]
*K*	Hydraulic conductivity	5.63 × 10^−12^ m^2^/(Pa s)	10^2^–10^11^ μm^3^ s/kg	[27]
*μ*	Dynamic viscosity	0.9 × 10^−3^ Pa s	–	[28, 29]
*ρ*	Interstitial fluid density	993.2 kg/m^3^	–	[30]
*ϕ*	Porosity	0.2		[31]
D*^b^	Solute diffusivity	–	10^1^–10^3^ μm^2^/s	[31, 19]
*l* ^a^	Resistance length	50; 100 μm		Model dependent
*h*	Resistance height	100 μm		Model dependent
*d*	Resistance depth	200 μm		Model dependent
*R*_*i*_^*PCY*^	Parenchyma inner radius	10 μm		[4]
*R*_*o*_^*PCY*^	Parenchyma outer radius	300 μm		Model dependent
*η*	Pore size	60 nm		[32]
*θ*	Phase shift	0	0–2π	Model dependent
*ξ*	Pulsatility ratio	0.80	0–1	[13]

^a^R2, R3 in the one vessel model and R6, R8, R9, R10, R11, R13, R15, R16, R17, R18, and R20 in the two vessel model had lengths of 100 μm. All others resistors had lengths of 50 μm except R5, R6, and R7 in the one vessel model which were defined as in Eq. 3

^b^D* refers to the effective solute diffusion coefficient in brain tissue

The parenchyma hydraulic resistance was derived by simplifying Darcy’s law for flow through rigid porous media to one-dimensional radial flow through a cylindrical shell.

3 $$R_{PCY} = \frac{{{ \ln }\left( {R_{o}^{PCY} /R_{i}^{PCY} } \right)}}{{2\pi hK_{PCY} }}$$

Here *R*_*o*_^*PCY*^, *R*_*i*_^*PCY*^, *h*, *K*_*PCY*_, are the outer and inner radii of the parenchymal cylindrical shell, the shell height, and the parenchyma hydraulic conductivity, respectively [33]. The outer radius of the parenchymal shell was taken as much larger than the inner radius to reflect the scale of the parenchyma theoretically available for flow.

Volumetric fluid sources were introduced into the network to account for fluid displaced by the arterial pulses in the cardiac cycle (Fig. 1a). No pressure gradients were imposed anywhere in the model and these volumetric fluid sources were the only drivers of fluid motion present. In-vivo measurements indicate that cortical vessel diameter variation in time is roughly sinusoidal [4]. An arterial wave speed of order 1 m/s [26] and pulse frequency of 5 Hz [14] correspond to a wavelength of 20 cm, much longer than the modeled 300 μm arteriole segment. It was therefore fair to assume a PVS inner radius that varies uniformly along its length [17] and sinusoidally in time. An expression for the rate of volume displacement due to uniform motion of the PVS inner boundary was found by differentiating the volume contained by the inner boundary with respect to time. Fluid volume displaced by the inner boundary moved into the PVS and parenchyma and appeared as a volumetric fluid source in the network model.

4 $$q = \dot{V} = 2\pi lr_{i} \dot{r}_{i}$$

Here *q*, *V*, *l*, and *r*_*i*_ are the volumetric flow rate, volume contained by the PVS inner boundary, the segment length modeled by the fluid source, and the PVS inner radius as a function of time, respectively.

The inner radius varied in time according to

5 $$r_{i} = - bcos\left( {2\pi f} \right) + R_{i}$$

Here *f* and *b* are the frequency and amplitude of inner wall motion, or the pulse frequency and amplitude. *R*_*i*_ is the time-averaged PVS inner radius value. Substituting Eq. 5 into Eq. 4 the flow rate became

6 $$q = 4{\pi ^2}lfb\left( {{R_i}\sin \left( {2\pi ft} \right) - b\sin \left( {2\pi ft} \right)\cos \left( {2\pi ft} \right)} \right)$$

Because the ratio of coefficients for the second and first term is *b*/*R*_*i*_, the first term dominates when *b* is much smaller than *R*_*i*_ and the flow rate is approximately

7 $$q \approx 4\pi^{2} lfbR_{i} \sin \left( {2\pi ft} \right)$$

Although the expression for PVS hydraulic resistance was derived for steady, axial pressure-driven flow, it serves as a reasonable approximation because the PVS thickness is much smaller than the pulse wavelength and the Womersley number, $\alpha = 2\left( {R_{o} - R_{i} } \right)\sqrt {2\pi f\rho /\mu }$, is small [34]. Twice the value of PVS thickness is the hydrodynamic radius [23] and *ρ* is the fluid density, approximately that of water at body temperature [30]. When PVS thickness is much smaller than wavelength, lubrication theory says radial velocity and pressure gradients can be assumed negligible, and axial velocity and pressure gradients dominate. When *α* is small, oscillatory flow can be approximated by the steady-state profile corresponding to the instantaneous axial pressure gradient in the segment [34]. The pulse amplitude was selected so that the free fluid hydraulic resistance of the PVS never varied by more than 5% and could be assumed constant when solving for pressure and velocity in the network.

To account for the presence of solid components in the PVS, an alternative resistance was derived by simplifying Darcy’s law for axial flow through an annulus of rigid porous media.

8 $$R_{PVS} = \frac{l}{{\pi (R_{o}^{2} - R_{i}^{2} )K_{PVS} }}$$

Here *l*, *R*_*o*_, *R*_*i*_, *K*_*PVS*_, are the PVS length modeled by the resistor, the PVS outer radius, the PVS inner radius, and the PVS hydraulic conductivity, respectively.

### Two vessel model

A planar portion of tissue which included segments of a cortical arteriole and vein, surrounding PVS and parenchyma were modeled as a network with 25 resistors (Fig. 1b). Vessels had a baseline radii of 10 μm [4] and were separated by 200 μm [19]. Fluid could enter or leave the network at the upper and lower boundaries of the modeled parenchyma and PVS. Because the flow produced by vessel pulsation was assumed to be radially symmetric, half of the radial flow produced by each vessel entered the modeled parenchyma and the flow rate for each arterial volumetric fluid source became.

9 $$q \approx 2\pi^{2} lfbR_{i} \sin \left( {2\pi ft} \right)$$

Accordingly, axial flow along half the PVS was modeled for the arteriole and the vein. The PVS resistances were therefore double those derived in the one vessel model because only half the annulus was available for flow.

The flow rate for each venous volumetric fluid source was determined by considering the pulsatility ratio between cortex arterioles and veins where pulsatility is defined as.

10 $$\Pi = 2\mathop \int \limits_{0}^{T} \left| {r_{i} - R_{i} } \right| dt$$

This formulation for pulsatility is based on Iliff et al. [13] where *T* is the measurement interval. Substituting Eq. 5 for inner radius variation over time into Eq. 10 revealed that pulsatility was proportional to pulse amplitude and inversely proportional to pulse frequency, Π = *b*/*πf*. The ratio of venous to arterial pulsatility, *ξ*, was used to determine the venous pulse amplitude for a given arterial pulse amplitude. Substituting the venous pulse amplitude into Eq. 9 produced the flow rate for each venous fluid source.

To assess the mode of solute transport in both the models, the Peclet number was computed for the PVS and parenchyma.

11 $$Pe = L_{PVS} v/D^{*}$$

12 $$Pe = L_{PCY} v/\phi D^{*}$$

13 $$Pe = \eta v/\phi D^{*}$$

Here *ϕ* and D* are the parenchyma porosity and solute diffusivity, respectively. The Peclet number formulation for the PVS, Eq. 11, includes *L*_*PVS*_, the full vessel segment length, and *v,* the average axial velocity. Two Peclet number formulations, Eqs. 12 and  13, were used for the parenchyma, differing in their characteristic length scale. The former includes *L*_*PCY*_, the distance between the arteriole and vein [19], and the latter includes *η*, an estimate of the parenchyma pore size [35].

Parameter sweeps were conducted to explore their effect on fluid motion in PVS and parenchyma. Parameters such as pulse amplitude, PVS inner and outer radius, and PVS and parenchyma hydraulic conductivity were varied for both the one vessel and two vessel models. In addition, the pulsatility ratio and pulse timing between arterial and venous pulses were varied in the two vessel model. Pulse timing was varied by adding a phase shift, *θ*, to the venous fluid production function.

14 $$q \approx 2\pi^{2} lfbR_{i} \sin \left( {2\pi ft - \theta } \right)$$

When a particular parameter(s) was varied the others remained at baseline values (Table 1) except in the PVS radii sweep where the pulse amplitude was reduced to 16.2 nm to account for PVS gap thicknesses as small as 1 μm without varying the PVS free-fluid hydraulic resistance by more than 5%

The authors use the term “oscillatory fluid motion”, “net fluid motion”, and “net flow” to refer to movement of fluid and reserve “solute transport”, “diffusion”, “dispersion,” and “convection” for the transport of solutes in the fluid medium. Oscillatory fluid motion is fluid motion that does not displace the mean position of the fluid over time unlike net fluid motion and net flow. Diffusion is the solute transport due to random molecular motion. Dispersion in this context is enhanced diffusion due to oscillatory fluid motion, and convection is solute transport along with a fluid undergoing net flow.

## Results

### One vessel model

**Fig. 2:**
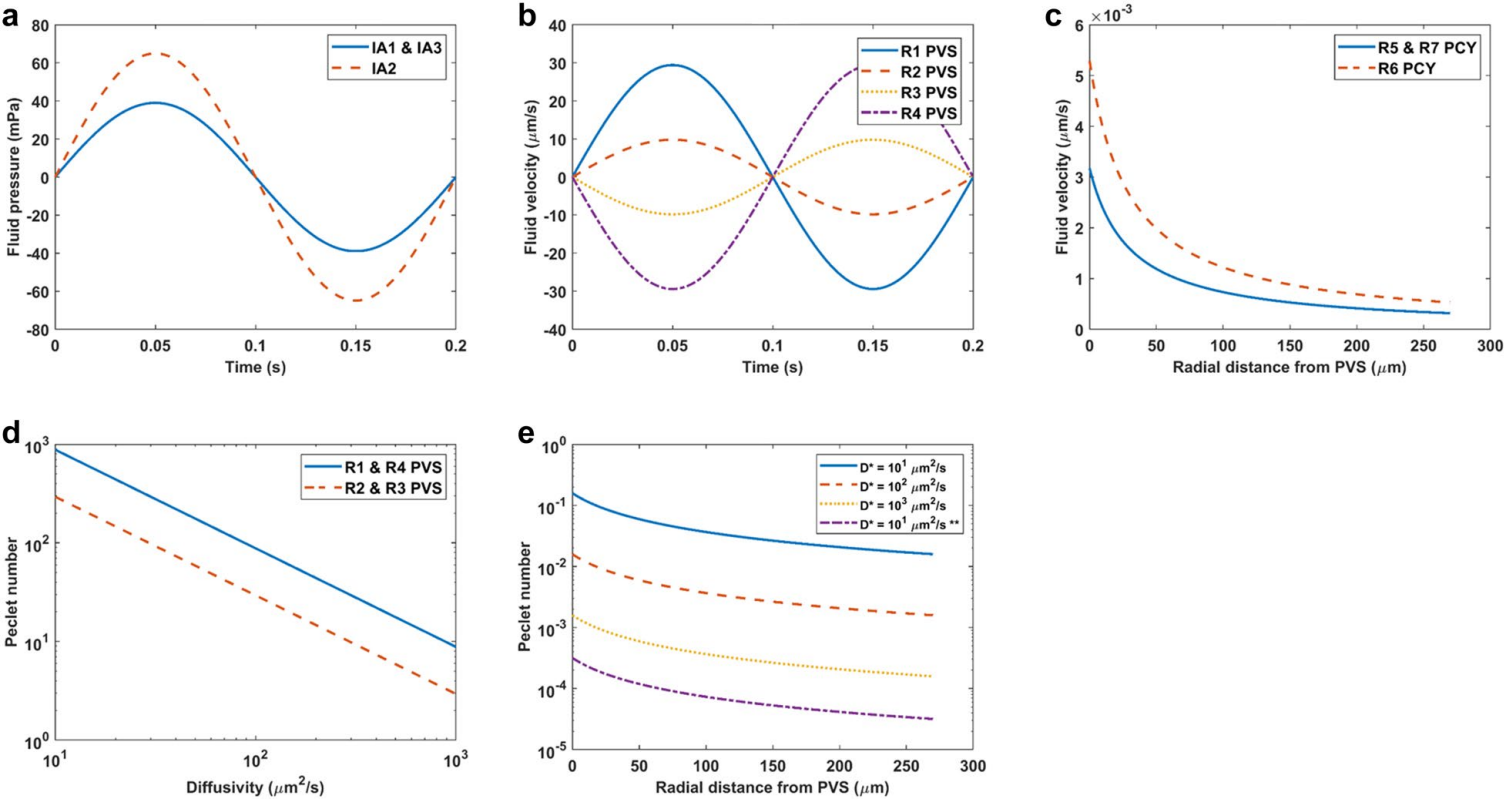
One vessel model baseline results. **a** Fluid pressure produced by volumetric fluid sources IA1, IA2, and IA3 over the course of one period. See Fig. 1 for source labels. **b** PVS fluid velocity over the course of one period for each PVS resistor. See Fig. 1 for resistor labels. **c** Parenchyma peak fluid velocity with distance from the PVS outer radius. **d** PVS Peclet numbers for a range of physiologically relevant diffusivities. **e** Parenchyma Peclet numbers with radial distance from the PVS outer radius for a range of physiologically relevant diffusivities. Peclet numbers were computed with the distance between vessels as the characteristic length (Eq. 12) for all diffusivities except that marked (**) for which pore size was the characteristic length (Eq. 13)

Cyclic variation in arteriole diameter in the one vessel model produced oscillatory fluid motion in both the PVS and parenchyma, but no net fluid motion (net flow) in any direction. Peak fluid velocity and pressure in the PVS were about 30 μm/s and 60 mPa, respectively (Fig. 2 a, b). Peak fluid velocity in the parenchyma close to the PVS was below 6 nm/s, and at a distance 50 μm from the PVS outer boundary decreased to less than 3 nm/s (Fig. 2 c). Peclet numbers for hypothetical solutes with diffusivities spanning 10–10^3^ μm^2^/s were mostly below 10^−1^ in the parenchyma indicating transport of physiological solutes there was diffusion dominated (Fig. 2e). In contrast, PVS Peclet numbers varied between 10^3^ and 10^1^ for the same span of diffusivities, suggesting physiological solute transport there had a convective component (Fig. 2d).

### Two vessel model

**Fig. 3:**
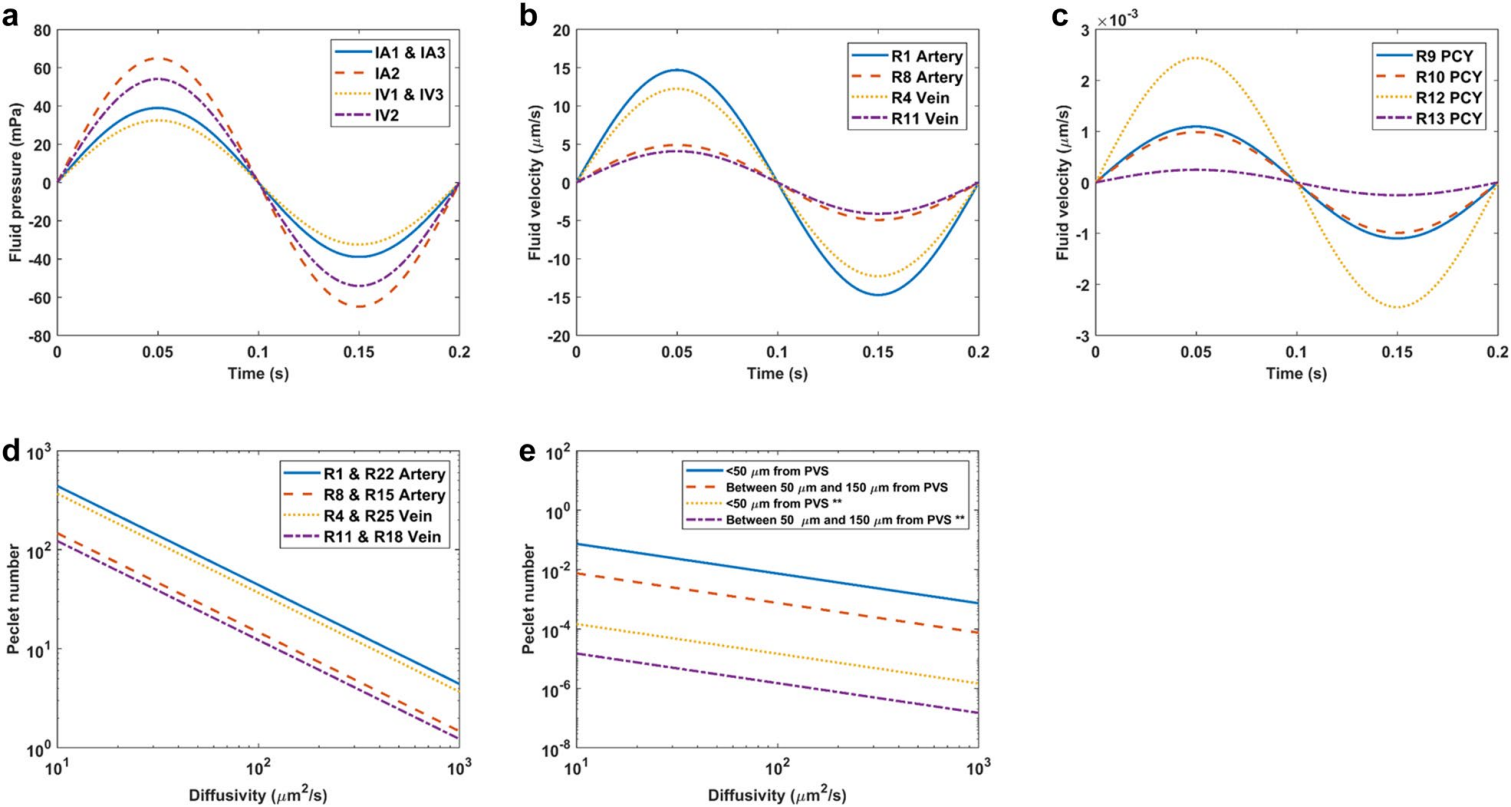
Two vessel model baseline results. **a** Fluid pressure produced by volumetric fluid sources over the course of one period. See Fig. 1 for source labels. **b** PVS fluid velocity over the course of one period. See Fig. 1 for resistor labels. **c** Parenchyma fluid velocity for resistors parallel and perpendicular to the vessels over the course of one period. **d** PVS Peclet numbers for a range of physiologically relevant diffusivities. **e** Parenchyma Peclet numbers with radial distance from the PVS outer radius for a range of physiologically relevant diffusivities. Peclet numbers were computed with the distance between vessels as the characteristic length (Eq. 12) for all cases except those marked (**) for which pore size was the characteristic length (Eq. 13)

Cyclic diameter variation in the arteriole and vein also produced oscillatory fluid motion in both the PVS and parenchyma, but no net fluid motion. For the baseline case, peak fluid velocity in the arterial PVS was approximately 15 μm/s, about half the peak velocity in the one vessel model, and peak pressure was 60 mPa which was similar to the one vessel model value (Fig. 3 a, b). Peak fluid velocity within the parenchyma was determined between 50 and 150 μm from the arterial PVS outer boundary, and it was found to be below 3 nm/s in both perpendicular and parallel directions to the vessels (Fig. 3c). Peak fluid velocity increased with proximity to the vessel which was in agreement with the one vessel model results (compare R12 and R13 in Fig. 3c). As in the one vessel model, Peclet numbers for hypothetical solutes with diffusivities spanning 10–10^3^ μm^2^/s were above 1 in the PVS (Fig. 3d) and below 10^−1^ in the parenchyma (Fig. 3e).

### Parameter sweeps

**Fig. 4:**
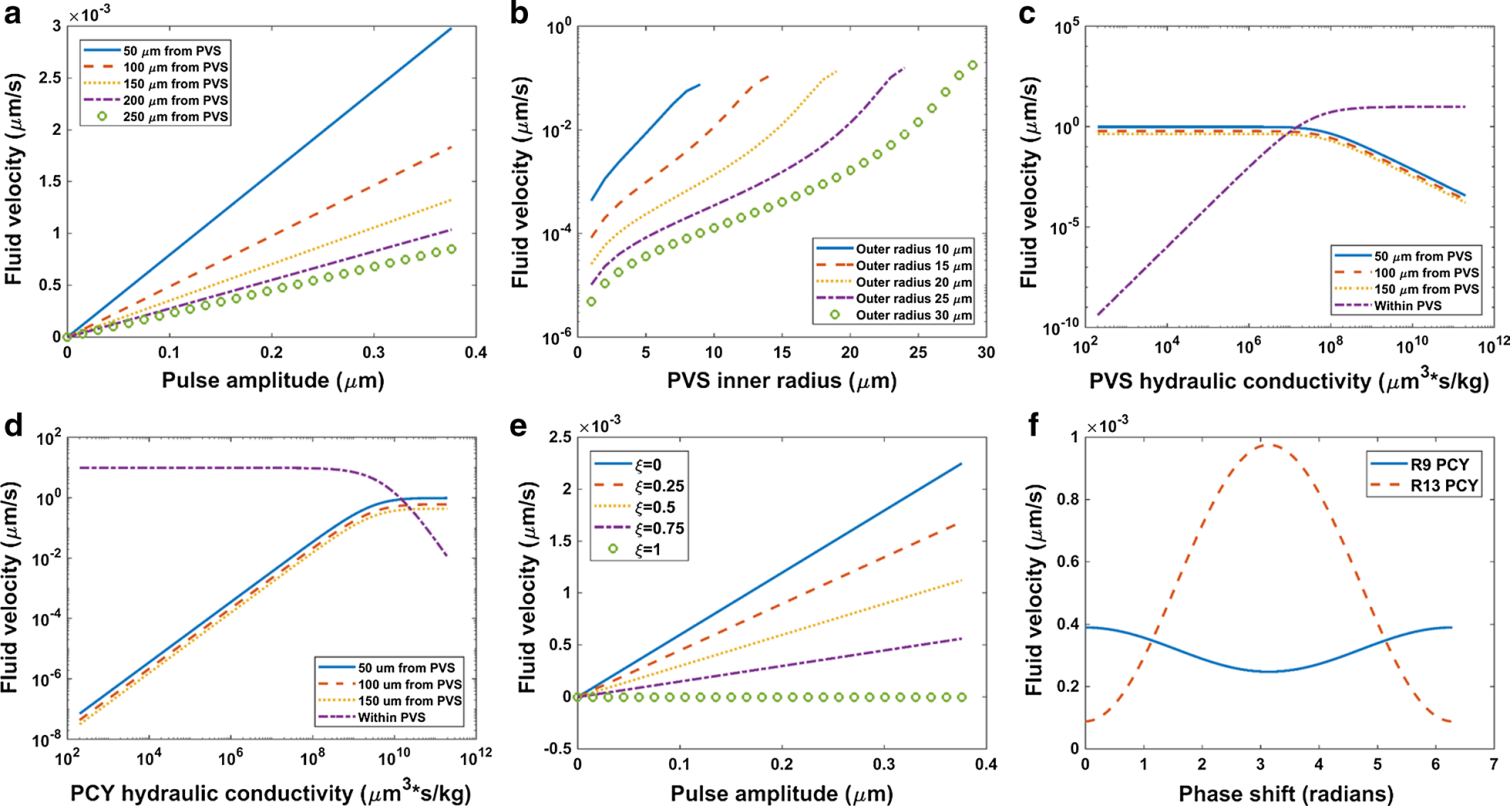
Effect of one vessel and two vessel model parameter sweeps on parenchyma peak fluid velocity. **a** One vessel model parenchyma peak fluid velocity (R6) as pulse amplitude varied for different radial distances from the PVS outer radius. See Fig. 1 for resistor labels. **b** One vessel model parenchyma peak fluid velocity (R6) as PVS inner radius varied for a range of outer radius values. **c** One vessel model PVS (R2) and parenchyma (R6) peak fluid velocity as PVS hydraulic conductivity varied. Here the porous media formulation for PVS hydraulic resistance was implemented (Eq. 8). **d** One vessel model PVS (R2) and parenchyma (R6) peak fluid velocity as parenchyma hydraulic conductivity varied. **e** Two vessel model parenchyma peak fluid velocity (R13) as pulse amplitude varied for a range of venous to arterial pulsatility ratios, ξ. **f** Two vessel model parenchyma peak fluid velocity (R13) as arterial and venous pulse timing (phase shift, $\theta$) varied

**Fig. 5:**
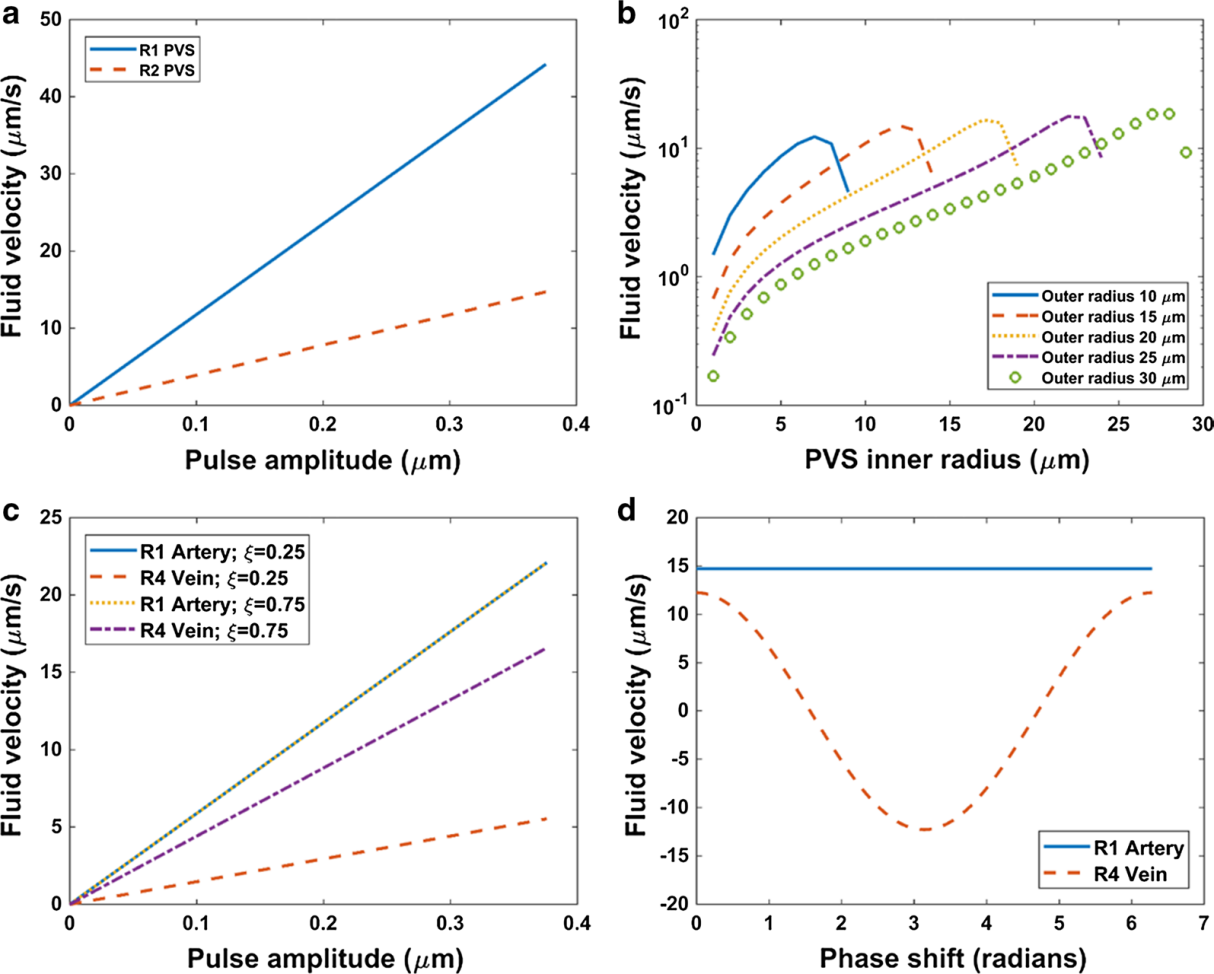
Effect of one vessel and two vessel model parameter sweeps on PVS fluid velocity. **a** One vessel model PVS peak fluid velocity as pulse amplitude varied. **b** One vessel model PVS peak fluid velocity as PVS inner radius varied for a range of outer radius values. Fluid velocity for R1 shown (see Fig. 1). **c** Two vessel model arterial and venous PVS peak fluid velocity as pulse amplitude varied for a range of venous to arterial pulsatility ratios, ξ. **d** Two vessel model arterial and venous PVS fluid velocity as arterial and venous pulse timing (phase shift, $\theta$) varied

In the one vessel model, peak fluid velocity in parenchyma increased linearly with pulse amplitude and decayed with distance from the PVS outer boundary (Fig. 4a). This velocity never exceeded 3 nm/s for the range of pulse amplitudes examined. Peak fluid velocity in the PVS also increased linearly with pulse amplitude and was greater near the ends of the PVS segment (Fig. 5a). For a given PVS outer radius, increasing the inner radius (without varying the pulse amplitude), increased peak fluid velocity in the PVS and parenchyma by several orders of magnitude (Fig. 4b, 5b). As the PVS became narrower, PVS resistance to flow increased, thus promoting flow into the parenchyma while restricting flow in the PVS. Peak fluid velocity in PVS and parenchyma varied non-linearly with changes in PVS inner and outer radii. Modeling the PVS as porous media revealed that as PVS hydraulic conductivity became unnaturally low the peak fluid velocity in parenchyma remained of order 1 μm/s. Alternatively, as PVS hydraulic conductivity approached that corresponding to a free fluid cavity (~ 10^10^ μm^3^ s/kg), peak fluid velocity in the parenchyma droped three orders of magnitude and fluid velocity in the PVS remained of order 10 μm/s (Fig. 4c) for R2 in the one vessel model. A similar pattern was also evident when parenchyma hydraulic conductivity was varied and the PVS was considered a free fluid cavity (Fig. 4d).

The two vessel model demonstrated a linear increase in parenchyma peak fluid velocity as pulse amplitude increased as in the one vessel model, but also showed that increasing the pulse amplitude difference between the arteriole and vein by decreasing venous pulsatility increased the peak fluid velocity in parenchyma perpendicular to the vessels (Fig. 4e). This decrease in venous pulsatility also decreased venous PVS peak fluid velocity but did not affect arterial PVS peak fluid velocity (Fig. 5c). Delaying the cyclic diameter variation of the vein with respect to the arteriole produced changes in parenchyma fluid velocity parallel and perpendicular to the vessels, but both velocities remained of order 10^−3^ μm/s at a distance of 50 μm from the arterial PVS outer boundary (Fig. 4f). Fluid velocity was measured a fourth period into the arterial fluid production waveform (Eq. 9). Arterial PVS fluid velocity was unaffected by this delay, but venous fluid velocity varied such that for some phase shifts arterial and venous PVS velocities were in opposite directions (Fig. 5d). The two vessel model followed similar trends as the one vessel model for variation in PVS radii and hydraulic conductivities (not shown).

## Discussion

Evidence has shown that transport of dissolved compounds in PVS cannot be explained by diffusion alone [3]. Consequently, convective solute transport by net flow through the PVS driven by vascular pulsatility has been forwarded as a rationale for rapid transport rates. This viewpoint is supported by evidence of reduced PVS uptake and clearance of compounds injected into CSF and parenchyma when vascular pulsatility is dampened [2, 13].

In the one vessel and two vessel models developed here, vascular pulsatility produced oscillating fluid motion in the PVS but did not produce net flow which is needed for convection to occur. As a result, it is more difficult to explain net solute uptake or clearance by convection. During vessel expansion, fluid moved out of the PVS segment through both ends. During vessel retraction, the flow direction was reversed such that no net flow was observed. This prediction aligns with previous observations of oscillatory tracer movement within PVS and computational predictions [5, 17]. Though no net flow was observed, the PVS Peclet numbers ranged between 1 and 10^3^ in the PVS (Fig. 2d; Fig. 3d) such that the fluid motion could promote solute transport by dispersion, as has been discussed previously [12, 17, 36]. Spatial variation in fluid velocity within the PVS may create temporary concentration gradients that enhance axial diffusion without net fluid flow. Dispersion could help explain discrepancies in transport direction through PVS seen in previous tracer uptake studies (influx into versus efflux from parenchyma) and the preference of solutes for arterial rather than venous PVS because of greater dispersion in the former [36].

The degree to which dispersion enhances axial diffusion for oscillating flow in a fluid filled annulus is proportional to the square of the volume displaced in each oscillation, also known as the tidal or stroke volume [37]. The tidal volume was greater in the arterial PVS than in venous PVS for the baseline case (Fig. 3b) and this difference grew with decreasing venous pulsatility (Fig. 5c). An increase in effective diffusion coefficient by up to a factor of two was previously predicted for solutes with diffusivities of 2 μm^2^/s for oscillating flow in a 250 μm PVS segment [17]. Given the average fluid velocity computed from their maximum flow rate (1590 μm/s) and cross-sectional area was less than the peak outlet velocity for arterial PVS reported here (30 μm/s) and that these predictions are likely underestimations that do not account for fluid volume displaced by vessel expansion downstream from the modeled segment, the dispersive effect could be greater still. PVS tapering likely influences PVS fluid motion and solute dispersion as well. As inner radius increased for a given outer radius, the volume displaced by the same pulse amplitude increased, and as outer radius decreased for a given inner radius, the PVS cross sectional area decreased both of which lead to an increase in fluid velocity except when the PVS gap thickness was small (Fig. 5b). Additional analysis of PVS branching networks is needed to determine the effect of downstream pulsatility and PVS tapering on flow velocity and dispersion within the PVS, especially when modeled as a porous media.

Both the one vessel and two vessel models predicted oscillatory fluid motion in the parenchyma but the peak fluid velocity was so small (≤ 6 nm/s) that the main solute transport mode was diffusion (Pe < 10^−1^) as in many other experiments and models [8, 9, 17–19]. Parenchyma fluid velocity of up to 16 nm/s and Peclet number of order 10^−1^ for a pressure difference of 1 mmHg/mm between arterial and venous PVS was recently predicted in a porous media computational model [19]. This fluid velocity is likely higher than that reported here because the pressure drop for the present baseline case is of order 10^−3^ mmHg/mm (Fig. 3a). Fluid velocity in the parenchyma increased with pulse amplitude (Fig. 4a), increasing pulse amplitude difference between the arteriole and vein (Fig. 4e), increasing PVS inner radius for a given outer radius, and decreasing PVS outer radius for a given inner radius (Fig. 4b) because of corresponding changes in volume displacement and PVS hydraulic conductivity. However, the parenchyma fluid velocity remained less than order 10^−1^ μm/s even for narrow PVS gap thicknesses. Variation in PVS and parenchyma hydraulic conductivity when PVS was considered a porous media indicated that even when PVS hydraulic conductivity was made to be unnaturally low, fluid velocity in the parenchyma was at most order 1 µm/s and decreased rapidly at high PVS hydraulic conductivity ranges (Fig. 4c). Computing Peclet number with pore size taken as the characteristic length as is often done in porous media [35] instead of the distance between the arteriole and vein suggests that even in these limiting cases, transport in parenchyma is expected to be diffusion dominated (Fig. 2e for baseline case). Parenchyma fluid velocity increased with increasing hydraulic conductivity as may be found along white matter tracts (Fig. 4d). Delaying the venous pulse relative to the arterial pulse did not produce changes in parenchyma fluid velocity large enough to affect this conclusion (Fig. 4f).

While the results show no net flow over time in the PVS (Fig. 2b; Fig. 3b), they do not rule out net flow produced by other phenomena not explicitly modeled such as time-varying PVS hydraulic conductivity [16, 38] and transient pressure differences between CSF and PVS spaces [38]. For example, a pressure gradient driving fluid into the PVS could be established when PVS hydraulic conductivity is high and a reversed gradient could be present when conductivity is low thus producing a net flow through PVS. This relies on timing differences between vascular and CSF pressure pulses [38]. Other drivers of net flow may include fluid exudation through the blood brain barrier at the capillary level [3, 12] and global pressure gradients responsible for CSF circulation. Capillary fluid production has been included as a global fluid source in previous convection enhanced drug delivery models [39, 40]. Net fluid movement could be established in an unverified, continuous arterial PVS to peri-capillary space to venous PVS path [2, 9], or an arterial PVS to parenchyma to venous PVS path [4]. The latter does not necessarily imply convective solute transport through parenchyma as proposed in glymphatic theory [4] because fluid velocity could be very low there (as expected) while maintaining net flow from arterial to venous PVS. However the magnitude, direction, and mechanical drivers of such net flows within PVS remain unclear. It is therefore important to quantify the degree to which dispersion via oscillatory flow due to vascular expansion can explain experimental solute transport in PVS, or if net flow caused by other factors must be present. It is even possible to imagine solute transport occurring down a concentration gradient opposite to the direction of net flow in the PVS if net flow is small relative to oscillatory flow. A distinguishing feature of solute transport by dispersion versus convection due to net flow is that the rate of the former varies with solute diffusivity [37] whereas the latter is independent of diffusivity. However, other complications to consider are tracer size-exclusion and the possibility of opposing flow directions within different regions of the PVS [7].

While the one and two vessel hydraulic resistance networks developed here are a coarse discretization of the flow domain they can nonetheless capture the effects of vessel diameter variation and tissue property changes on fluid motion within the PVS and parenchyma simultaneously. Because the parenchyma was modeled as rigid porous media, these models did not capture parenchyma deformation expected to accompany vessel volume change in vivo which might result in unsteady variation in PVS hydraulic conductivity. Non-linear, viscoelastic tissue properties might play a role in producing net fluid motion as hydraulic conductivity could vary with unsteady deformation rates during the cardiac cycle. CFD models that account for interaction between fluid–solid interfaces and viscoelastic tissue properties would provide further insight into fluid motion and solute transport.

## Conclusions

Two hydraulic network models were developed to predict the fluid motion produced by blood vessel pulsations in PVS and parenchyma. Periodic changes in vessel volume resulted in oscillatory fluid motion in PVS and parenchyma but no net flow over time. Peclet numbers indicated solute transport is diffusion dominated in parenchyma but might be enhanced by dispersion in PVS. Peak fluid velocity in the PVS tended to increase with increasing pulse amplitude and vessel size. While these results to do not rule out possible net flow in the PVS due to unsteady PVS hydraulic resistance and non-linear tissue properties, they do encourage further investigation into dispersion as an alternative mechanism for rapid solute transport in PVS.

## Abbreviations

PVS: perivascular space(s)
SAS: subarachnoid space(s)
ISF: interstitial fluid
CSF: cerebrospinal fluid
CFD: computational fluid dynamics
PCY: parenchyma
R#: resistance number
IA#: arterial source number
IV#: venous source number
Pe: Peclet number
